# Sidewall Slope Control of InP Via Holes for 3D Integration

**DOI:** 10.3390/mi12010089

**Published:** 2021-01-16

**Authors:** Jongwon Lee, Kilsun Roh, Sung-Kyu Lim, Youngsu Kim

**Affiliations:** 1 Division of System IC Development, National Nanofab Center, Daejeon 34141, Korea; 2 Division of Nano Patterning Process, National Nanofab Center, Daejeon 34141, Korea; ksnoh@nnfc.re.kr; 3 Division of Nano Thin Film Process, National Nanofab Center, Daejeon 34141, Korea

**Keywords:** InP, via hole, 3D integration, through substrate via (TSV)

## Abstract

This is the first demonstration of sidewall slope control of InP via holes with an etch depth of more than 10 μm for 3D integration. The process for the InP via holes utilizes a common SiO_2_ layer as an InP etch mask and conventional inductively coupled plasma (ICP) etcher operated at room temperature and simple gas mixtures of Cl_2_/Ar for InP dry etch. Sidewall slope of InP via holes is controlled within the range of 80 to 90 degrees by changing the ICP power in the ICP etcher and adopting a dry-etched SiO_2_ layer with a sidewall slope of 70 degrees. Furthermore, the sidewall slope control of the InP via holes in a wide range of 36 to 69 degrees is possible by changing the RF power in the etcher and introducing a wet-etched SiO_2_ layer with a small sidewall slope of 2 degrees; this wide slope control is due to the change of InP-to-SiO_2_ selectivity with RF power.

## 1. Introduction

Owing to the excellent electron mobility characteristic of InAs, InGaAs, and InAlAs materials monolithically grown on InP substrate, InP-based electrical semiconductor device technologies such as high electron mobility transistors (HEMT), heterojunction bipolar transistors (HBT), and resonant tunneling diodes (RTD) have been demonstrated with maximum oscillation frequencies exceeding one terahertz [1,2,3]. To optimally exploit these high-frequency InP devices in system applications for millimeter-imaging and wideband communication, InP technologies have been integrated with Si or GaN technologies, which play a role of peripheral ICs or high-power ICs, respectively. Of various integration technologies, three-dimensional (3D) integration technologies have recently been demonstrated; compared to two-dimensional (2D) technologies, these 3D technologies minimize the resistance of interconnect-lines and improve chip density [4,5,6].

InP-based 3D integration technology has a core process called through-substrate-via (TSV), which forms via holes in the InP substrate and fills them with low-resistivity metals [6]. Similar to the Si TSV process, the InP TSV process has been realized by performing metal deposition on InP via holes followed by chemical-mechanical-polishing (CMP) [6]. However, the InP CMP process has to be handled carefully due to the fragile nature of InP substrates and equipment for InP CMP is uncommon and difficult for researchers to access. Instead of the CMP-based TSV process, an electro-plating (EP)-based TSV process, which performs seed metal deposition on InP via holes followed by EP, can be utilized. While the CMP-based TSV process completely fills via holes with metals, the EP-based process deposits thin metals with a thickness of only a few micrometers (μm) on the edges of the InP via holes. Because a few-μm-thick edge-deposited metal layers based on Cu or Au do not degrade the interconnect resistance of InP TSV because the skin depth, given by 1/(πfμσ)^2^ [7], where f is the operating frequency, μ is the relative permeability and σ is the conductivity, of low resistivity metals such as Cu or Au is calculated to be less than 0.5 micrometer (μm) in millimeter-wave frequency range of more than 30 GHz, the EP-based TSV process has recently been utilized as a useful hole filling method for InP TSV [8].

**Figure 1:**
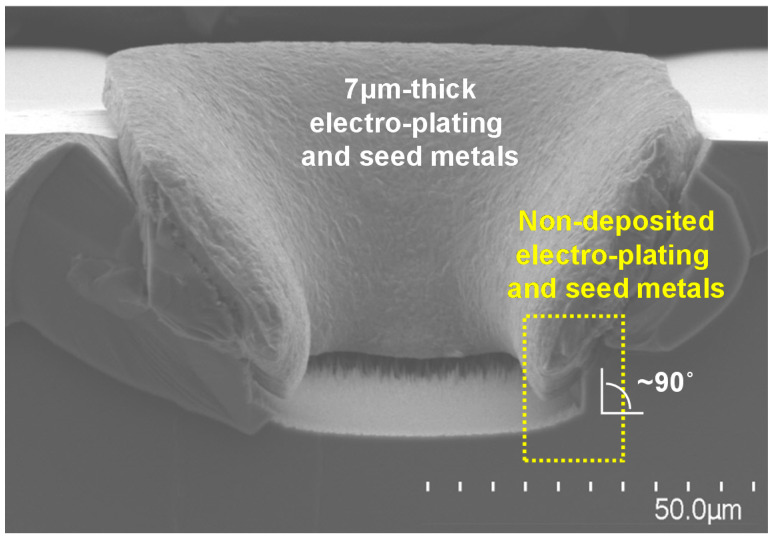
Scanning electron microscope (SEM) images of InP via holes with vertical sidewall slope where electro-plating and seed metals were deposited. Sputtering process for seed metal deposition was performed by using equipment of SRN-110 (SORONA, Anseong, Korea) with a substrate-rotation function.

Because sputter equipment has better step coverage than that of evaporator equipment, seed metals in the EP-based InP TSV process have mainly been deposited mainly by sputtering. However, sputter-based seed metal deposition is known to suffer from shadowing effects when the aspect ratio (AR) of via holes, defined as the ratio of the height to the width of the structures, is more than one, leading to poor step coverage on via hole sidewalls [9]. As shown in our experimental results in Figure 1, it was revealed that Ti/Cu EP and seed metals on a sidewall of InP via holes with vertical slope of 90 degrees were not deposited. Accordingly, process methodology for controlling the sidewall slope of InP via holes should be presented so that they InP via holes have gradual sidewall slope. To date, most reports have focused only on implementing InP structures with vertical sidewall slopes approaching 90 degrees [10,11,12,13,14,15,16].

In this paper, we propose for the first time a process methodology for sidewall slope control of InP via holes. The etch depth of InP via holes is targeted to exceed 10 μm, because the depth of TSV for 3D integration has been decreased to about 10 μm, which corresponds to the limit value of the CMP processes [6,7]. The process for InP via holes with an etch depth of more than 10 μm utilizes a common SiO_2_ layer as an InP etch mask and conventional inductively coupled plasma (ICP) etcher and simple gas mixtures of Cl_2_/Ar for InP dry etch. By controlling input parameters for the ICP etcher, such as ICP power, RF power and gas mixture ratio, and changing the sidewall slope of the SiO_2_ layer, the sidewall slope of InP via holes is possible to adjust in a wide range of 36 to 90 degrees, while retaining high InP etch rate of 1 μm/min. 

## 2. Materials and Methods

**Figure 2:**
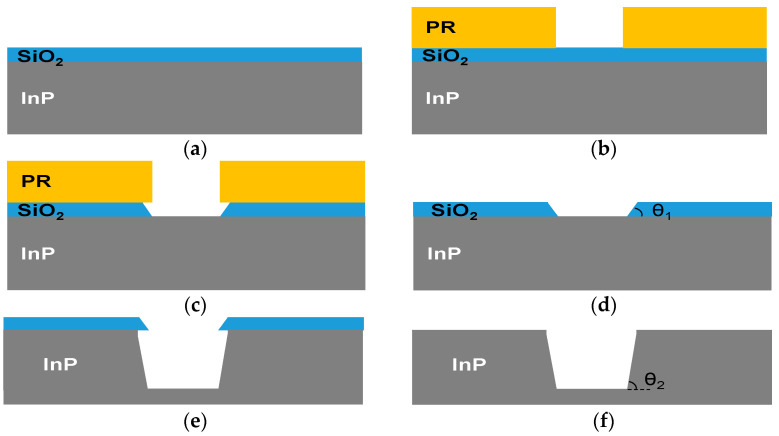
Cross-sectional view of a process flow for InP via holes: (**a**) SiO_2_ deposition on InP substrate; (**b**) formation of photoresist (PR) mask; (**c**) SiO_2_ patterning through the PR mask; (**d**) PR removal; (**e**) dry etching of the InP substrate; (**f**) SiO_2_ removal. θ_1_ and θ_2_ are defined as sidewall slopes of the SiO_2_ layer and InP substrate, respectively.

Figure 2 shows a cross-sectional view of a process flow for InP via holes. An InP substrate with S-doped, n-type and 100 orientation was used, provided by JX Nippon Mining & Metal Corporation. As shown in Figure 2a, a 1 μm thick SiO_2_ layer on the InP substrate was deposited by plasma-enhanced chemical vapor deposition (PECVD) at a temperature of 300 °C to use as an etch mask for InP dry etching. As shown in Figure 2b, a photoresist (PR) mask was formed by photolithography process with conditions of spinning rate of 3000 RPM, soft bake of 90 °C and 90 s, exposure time of 6 s, developing time of 50 s and hardbake of 150 °C and 15 min. The SiO_2_ layer was patterned by dry etching or wet etching process through the PR mask, as shown in Figure 2c. Dry etching process for the SiO_2_ layer was performed using an ICP etcher (Oxford Instruments Plasma Technology Plasmalab System 100) with conditions of CHF_3_/Ar of 20/10 sccm, ICP/RF power of 1500/300 W, operating pressure of 10 mTorr and operating time of total 15 min (5 times at 3 min a time). Wet etching process for the SiO_2_ layer was carried out by dipping the sample for 2.5 min in diluted HF (DHF) solution of HF:H_2_O = 1:3 with an SiO_2_ etch rate of 450 nm/min. The PR mask on the patterned SiO_2_ layer was removed using a DPSS-2200 solution, as shown in Figure 2d. The sidewall slope of the dry-etched or wet-etched SiO_2_ layers was defined as θ_1_, as can be seen in Figure 2d. Native oxide layer on the InP substrate was eliminated using a diluted HCl solution of HCl:DI = 1:10. The InP dry etching process was performed by using the ICP etcher, as shown in Figure 2e, and the SiO_2_ layer was removed by the DHF solution, as shown in Figure 2f. The sidewall slope of InP via holes was defined as θ_2_, as can be seen in Figure 2f.

The operation pressure, the temperature, and the type of the gas mixture are important input parameters of the ICP etcher for the InP dry etching process that determine the etch morphology of the InP via holes. Because the plasma environment in the etcher did not stably form at operating pressures of less than 4 mT, and because InP etch rate of InP via holes decreased considerably at operating pressures of more than 7 mT, the operating pressure in the ICP etcher for the InP dry etching was fixed at 5 mT. Because high operation temperature is known not to be necessary to achieve high InP etch rate of InP via holes when the reactive species densities are high [11,14], the operation temperature of the etcher for InP dry etching was set at room temperature while maintaining ICP power higher than 1000 W. CH_4_-based gas mixtures demonstrated slow InP etch rate and polymer deposition issues [11], and so chlorine (Cl_2_)-based gas mixtures of Cl_2_/Ar were used in this work to achieve high InP etch rate of InP via holes.

Each fabrication run consisted of 14 samples with a size of 2 cm × 2 cm. Twenty InP via holes with the same layout width of 10 μm were arranged in each sample. Different InP etching conditions were applied to each sample, by varying the RF power, gas mixture ratio, and ICP power of the ICP etcher for the InP dry etching process. A total of three fabrication runs were carried out to identify parameter deviation caused by process variation. The etching morphology of the fabricated InP via holes was observed using scanning electron microscope (SEM) equipment of S-4800 (Hitachi, Ltd., Tokyo, Japan). 

## 3. Results and Discussion

### 3.1. InP via Holes with Steep Sidewall Slopes of 80 to 90 Degrees

**Figure 3:**
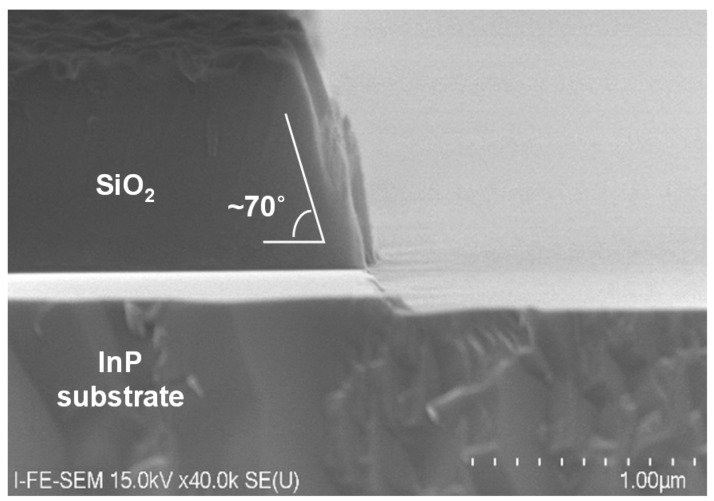
SEM images near a sidewall of a dry-etched SiO_2_ layer patterned on InP substrate.

To implement InP via holes with steep sidewall slope of more than 80 degrees, a dry-etched SiO_2_ layer with an average θ_1_ of 70 degrees as an etch mask for InP dry etching was utilized, as shown in Figure 3.

**Figure 4:**
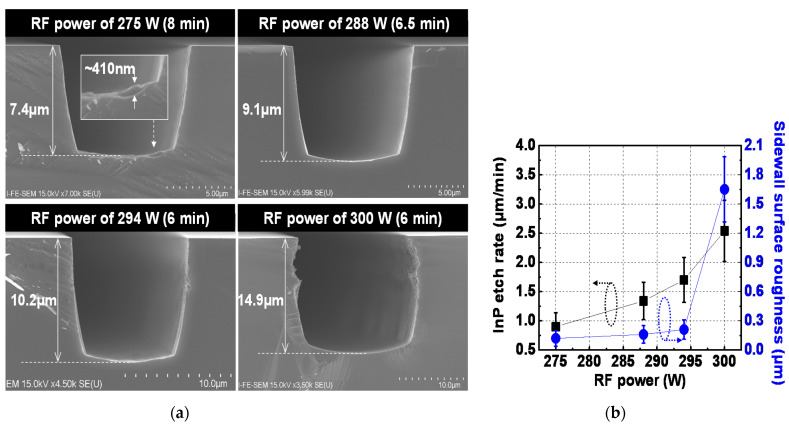
Fabrication results of InP via holes etched at different levels of RF power: (**a**) SEM images of representative etch profiles and (**b**) InP etch rate and sidewall surface roughness of InP via holes. Error bars in Figure 4b represent the deviation values incurred by the process variation for a total of 60 InP via holes with the same layout width in the three fabrication lots. ICP power, Cl_2_/Ar gas mixture, operation pressure, and operation temperature of the ICP etcher were 1200 W, 20/15 sccm, 5 mT and room temperature, respectively.

An experiment to determine the appropriate value of RF power in the ICP etcher for InP dry etching was performed. The fabrication results of InP via holes etched at different levels of RF power of 275 W, 288 W, 294 W, and 300 W are shown in Figure 4, where Figure 4a,b show SEM images of representative etch profiles and the InP etch rate and sidewall surface roughness of the fabricated InP via holes, respectively. Error bars in Figure 4b represent the deviation values incurred by the process variation for a total of 60 InP via holes with the same layout width in the three fabrication lots. ICP power and Cl_2_/Ar gas mixture of the ICP etcher were fixed at 1200 W and 20/15 sccm, respectively. An InP via hole with an RF power of 300 W showed a bad sidewall profile, as can be seen in Figure 4a, exhibiting a high average sidewall surface roughness of 1.65 μm, as shown in Figure 4b. This bad profile phenomenon is attributed to the strong Ar-ion bombardment near the top of the InP via hole by the high electric field. As RF power decreased, InP etch rate showed a decreasing tendency and, especially, average InP etch rate at RF power of 275 W decreased to 0.9 μm/min, as shown in Figure 4b. From our experimental results, performing InP dry etching at once rather than carrying out it by dividing several times led to much less polymer deposition on the surfaces of InP via holes. When InP dry etching is performed at once, unintended abnormal behavior has occurred in case of processing time of the ICP etcher exceeding 10 min; there was a problem of redeposition of various polymers on the surface of the chamber due to the rise in temperature in the chamber. Therefore, the processing time of the ICP etcher for the InP dry etching must be limited to within 10 min; for this reason, to implement InP via holes with an etch depth of more than 10 μm in 10 min, the InP etch rate had to exceed 1 μm/min. Consquently, the correct value of RF power to form InP via holes with steep sidewall slope was in the range of about 280 W to 294 W. The sidewall roughness of InP via holes formed with the RF power values of 280–294 W was at most 310 nm. Because seed metals with thickness of more than 310 nm can be readily deposited using sputter equipment, the achieved roughness value of 310 nm does not have a significant effect on the performance of the EP-based InP TSV with metal thicknesses of a few-μm level. Regarding the roughness value of 310 nm, it was analyzed that 210 nm of this value is generated from the InP dry etching process because 100 nm of that value is the sidewall roughness of the dry-etched SiO_2_ layer itself, as can be seen in Figure 3.

**Figure 5:**
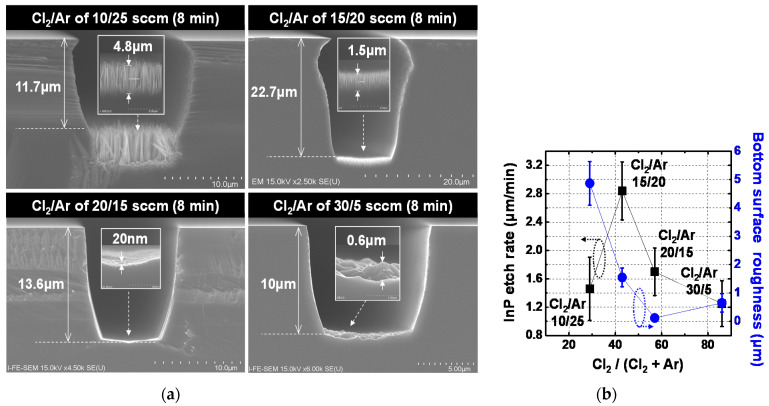
Fabrication results of InP via holes etched at Cl_2_/Ar gas mixtures with different ratio of 10/25, 15/20, 20/15, and 30/5 sccm: (**a**) SEM images of representative etch profiles and (**b**) InP etch rate and bottom surface roughness of fabricated InP via holes. Error bars in Figure 5b represent the deviation values for a total of 60 InP via holes with the identical layout pattern in the three fabrication lots. Total gas flow rate, ICP power, RF power, operation pressure, and operation temperature of the ICP etcher were 35 sccm, 1200 W, 294 W, 5 mT and room temperature, respectively.

The proper ratio for the gas mixture of Cl_2_/Ar was investigated. Figure 5 shows fabrication results for InP via holes etched at mixtures of 10Cl_2_/25Ar, 15Cl_2_/20Ar, 20Cl_2_/15Ar, and 30Cl_2_/5Ar, while fixing total gas flow rate at 35 sccm. Figure 5a,b show SEM images of representative etch profiles and the InP etch rate and bottom surface roughness of the fabricated InP via holes, respectively. Error bars in Figure 5b indicate the deviation values for a total of 60 InP via holes with the identical layout width in the three fabrication lots. ICP power and RF power of the ICP etcher were fixed at 1200 W and 294 W, respectively. Fabricated InP via holes with Ar-rich gas mixtures of 10Cl_2_/25Ar and 15Cl_2_/20Ar exhibited undercut profiles, as shown in Figure 5a, leading to seed metals not deposited on InP via hole, and rough bottom surface morphology, showing an average bottom surface roughness of 1.55 μm even at a slight Ar-rich mixture of 15Cl_2_/20Ar, as shown in Figure 5b. These bad morphologies are attributed to P deficiency phenomena in InP near the top of the sidewall and at the bottoms of InP via holes due to strong Ar-ion bombardment in the ICP etcher [17]. Compared to the Ar-rich mixtures-based InP via holes, Cl_2_-rich mixture-based InP via holes of 20Cl_2_/15Ar and 30Cl_2_/5Ar exhibited improved morphology without undercut shape or rough bottom surface issues, as shown in Figure 5a. The average bottom surface roughness of 0.65 μm for 30Cl_2_/5Ar is considered to result from the lack of physical desorption of chlorine-based etch products due to the lack of Ar-ion bombardment. As a result, gas mixture of 20Cl_2_/15Ar was selected to implement the InP via holes with steep sidewall slope, leading to InP via holes with bottom surface roughness of maximum 170 nm while maintaining an InP etch rate of more than 1 μm, as shown in Figure 5b.

**Figure 6:**
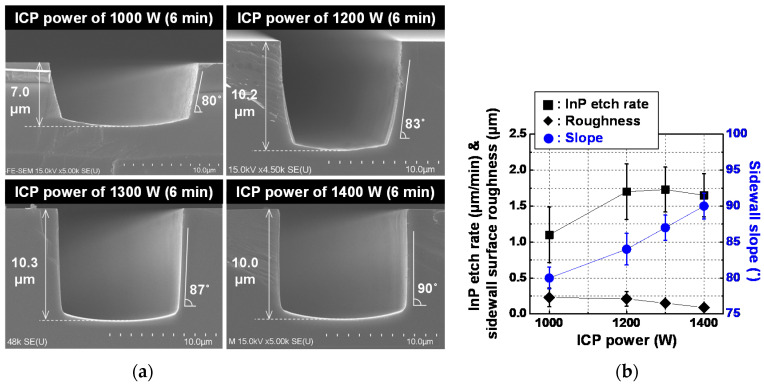
Fabrication results of InP via holes etched at different levels of ICP power: (**a**) SEM images of representative etch profiles and (**b**) InP etch rate, sidewall surface roughness, and sidewall slope of fabricated InP via holes. Error bars in Figure 6b represent the deviation values for a total of 60 InP via holes with the identical layout pattern in the three fabrication lots. RF power, Cl_2_/Ar gas mixture, operation pressure and operation temperature of the ICP etcher were 294 W, 20/15 sccm, 5 mT and room temperature, respectively.

With selected conditions of RF power of 294 W and gas mixture of 20Cl_2_/15Ar, various sidewall slopes of InP via holes with steep sidewall slopes of more than 80 degrees were achieved by careful control of the ICP power. Fabrication results for InP via holes etched at different levels of ICP power is shown in Figure 6, where Figure 6a,b show SEM images of representative etch profiles and the InP etch rate, sidewall surface roughness and sidewall slope of the fabricated InP via holes, respectively. Error bars represent the deviation values for a total of 60 InP via holes with the identical layout width in the three fabrication lots. ICP power of more than 1000 W was required to obtain InP etch rate of more than 1 μm/min. Average θ_2_ values of InP via holes changed almost linearly to 80, 84, 87, and 90 degrees at ICP power values of 1000, 1200, 1300, and 1400 W, respectively. It is considered that this change of the sidewall slope with ICP power originated from the increase of ion-assisted desorption on the sidewall surface of InP via holes due to the increase of ICP power. As the ICP power increased from 1000 W to 1400 W, the average sidewall surface roughness of the InP via holes improved from 230 nm to 90 nm, while maintaining the average InP etch rate of more than 1 μm/min, as shown in Figure 6b. For reference, the selectivity between the InP substrate and the SiO_2_ layer (InP-to-SiO_2_ selectivity), defined as the InP etch rate divided by SiO_2_ etch rate, showed average values of 12.5, 15.2, 15, and 13.3 at ICP power values of 1000, 1200, 1300, and 1400 W, respectively.

### 3.2. InP via Holes with Gradual Sidewall Slopes of 36 to 69 Degrees

**Figure 7:**
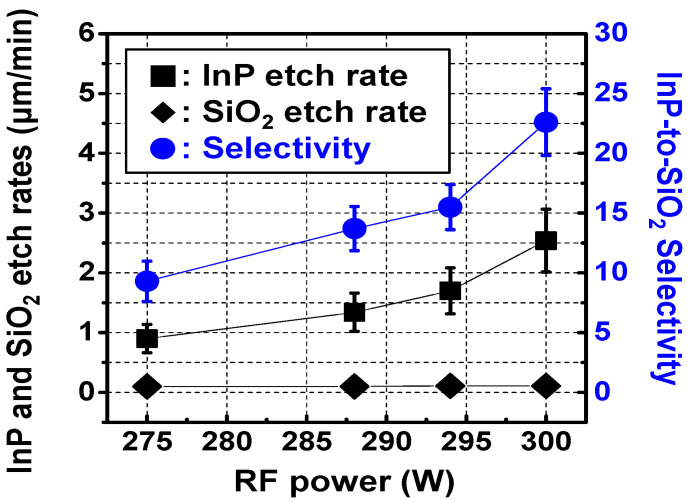
Selectivity between the InP substrate and the SiO_2_ layer (InP-to-SiO_2_ selectivity) of InP via holes etched at different levels of RF power. Error bars represent the deviation values for a total of 60 InP via holes with the identical layout pattern in the three fabrication lots.

InP-to-SiO_2_ selectivity was analyzed by re-visiting the experimental results of InP via holes etched at different levels of RF power, mentioned in chapter 3.1. Figure 7 shows InP-to-SiO_2_ selectivity as a function of RF power. As RF power increased from 275 W to 300 W, average InP-to-SiO_2_ selectivity increased from 9.3 to 22.6. This increase in selectivity occurred because average InP etch rate increased from 0.9 to 2.54 μm/min, while the average SiO_2_ etch rate remained constant in a range of 0.10 to 0.11 μm/min. We utilized this characteristic of InP-to-SiO_2_ selectivity versus RF power for slope control of InP via holes with gradual sidewall slopes.

**Figure 8:**
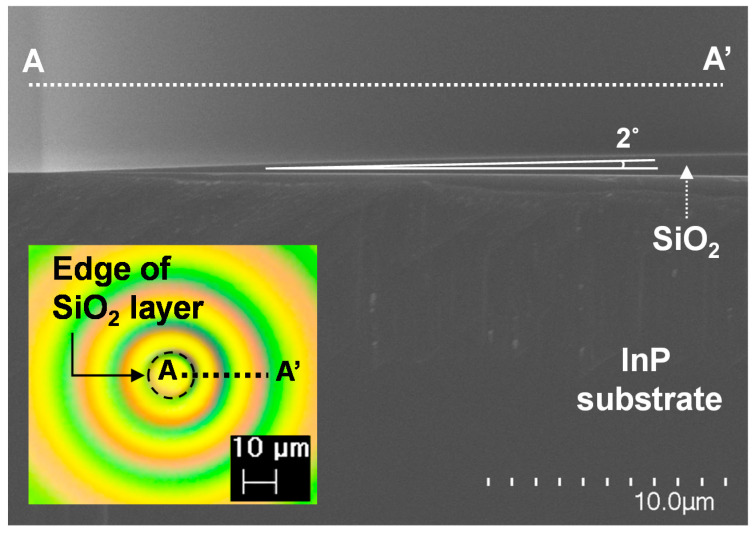
SEM image near a sidewall of a wet-etched SiO_2_ layer patterned on InP substrate. Inset indicates a floor plan image of the patterned wet-etched SiO_2_ layer by microscope. Dotted circle in the inset marks the edge of the patterned SiO_2_ layer.

To realize InP via holes with gradual sidewall slopes, a SiO_2_ hard mask layer with fairly small θ_1_ values of only a few degrees was required. Table 1 shows specific process flow and measured results for the SiO_2_ layer with a small slope. Instead of dry etching, a wet-etching process was selected to implement the SiO_2_ layer with gradual sidewall slopes. To identify an appropriate etchant for SiO_2_ wet etching, a 20:1 buffered oxide etch (BOE) solution with a mixture ratio of NH4F:HF = 38.1:2.4% and a DHF solution with a mixture ratio of HF:H_2_O = 1:3 were tested. Under the same hard bake process conditions of 120 °C and 15 min, the SiO_2_ layers wet-etched using the DHF solution exhibited an average θ_1_ of 2.55 degrees, while the SiO_2_ layers wet-etched using the BOE solution showed an average θ_1_ of 44.25 degrees. Because the θ_1_ value of 44.25 degrees of the BOE-based SiO_2_ layers was too large to implement InP via holes with gradual sidewall slopes when considering the above-mentioned values of InP-to-SiO_2_ selectivity, the DHF solution was chosen as an appropriate etchant for SiO_2_ wet etching. This smaller sidewall slope characteristic of the SiO_2_ layers based on the DHF solution compared to the BOE solution stems from the strong lateral etching property of concentrated HF. In addition, the hard bake conditions of baking temperature and baking time in the photo-lithography for the DHF-based SiO_2_ layers were optimized. At hard bake conditions of 120 °C and 5 min, there was a PR adhesion problem, where PR peeled from the SiO_2_ layers during the wet-etching process. By increasing the baking temperature from 120 °C to 150 °C while keeping the baking time above 15 min, the SiO_2_ layer wet-etched using the DHF exhibited a reproducible sidewall slope characteristic, demonstrating an average θ_1_ value and θ_1_ deviation of 2 degrees and ±5%, respectively, without a PR adhesion problem, as shown in Figure 8. The thickness of about 1 μm of the SiO_2_ layer was well maintained within ±2.7% deviation by using a PECVD equipment (SLR-730, UNAXIS, OC Oerlikon, Pfäffikon, Switzerland) operated at 300 degrees.

**Figure 9:**
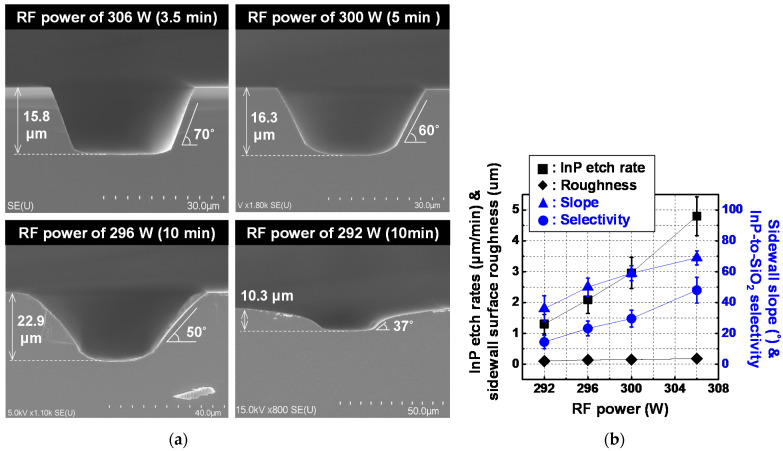
Fabrication results of InP via holes etched at different levels of RF power using a wet-etched SiO_2_ layer as an etch mask for InP dry etch: (**a**) SEM images of representative etch profiles and (**b**) InP etch rate, sidewall surface roughness, sidewall slope and InP-to-SiO_2_ selectivity of InP via holes. Error bars in Figure 9b represent the deviation value incurred by process variation for a total of 60 InP via holes with the identical layout pattern in the three fabrication lots. ICP power, Cl_2_/Ar gas mixture, operation pressure, and operation temperature of the ICP etcher were 1200 W, 18/17 sccm, 5 mT, and room temperature, respectively.

Figure 9 shows fabrication results for InP via holes etched at different levels of RF power using the wet-etched SiO_2_ layer, while fixing Cl_2_/Ar gas mixture and ICP power as 18/17 sccm and 1200 W, respectively. Figure 9a,b show SEM images of representative etch profiles and the InP etch rate, sidewall surface roughness, InP-to-SiO_2_ selectivity and sidewall slope of the fabricated InP via holes, respectively. Error bars represent the deviation values for 60 InP via holes with the identical layout width in the three lots. At values of RF power of 292, 296, 300, and 306 W, InP via holes exhibited average values of sidewall slope of 36, 50, 59, and 69 degrees, respectively, while maintaining InP etch rates of more than 1 μm/min. The two graphs of sidewall slope and InP-to-SiO_2_ selectivity as a function of RF power showed similar tendency, as shown in Figure 9b, which proves that this change of sidewall slope is attributed to the change of InP-to-SiO_2_ selectivity with RF power. For reference, surface roughness in the InP via holes with gradual sidewall slopes was at most 230 nm.

**Figure 10:**
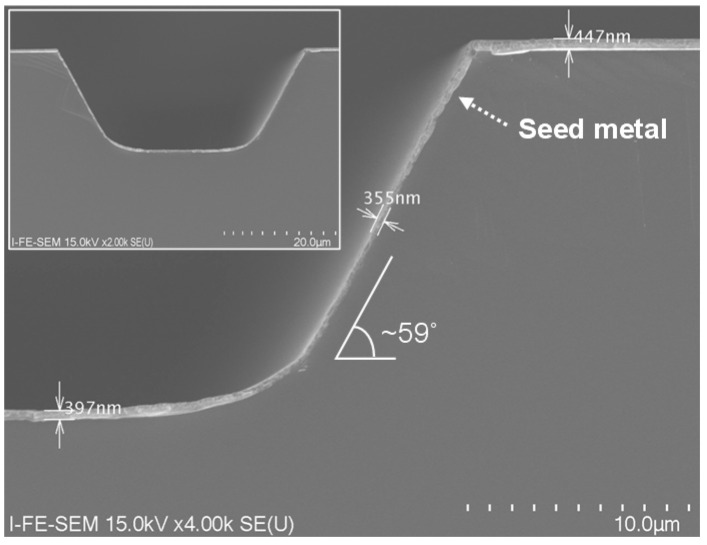
SEM images of an InP via hole with a gradual sidewall slope of 59 degrees where seed metals were deposited.

We should note that the proposed process method for obtaining gradual-sloped InP via holes leads to a penalty in area as an opportunity cost in providing the slope control technique. Table 2 shows a footprint comparison between the InP via holes with steep sidewall slopes and the InP via holes with gradual sidewall slopes. The footprint width (W_FOOT_) and footprint ratio (R_FOOT_) denote the average hole width of the top side of InP via holes and the W_FOOT_ ratio of InP via holes with gradual sidewall slopes over InP via holes with steep sidewall slopes, respectively. The W_FOOT_ value of the steep-sloped InP via holes was measured to be 11.5 μm and the W_FOOT_ values of the gradual-sloped InP via holes with average θ_2_ values of 69, 59, 50, and 36 degrees were 36, 39.2, 62.4, and 101.3 μm, respectively, resulting in corresponding R_FOOT_ values of 3.1, 3.4, 5.4, and 8.8. From our experimental results, it was verified that sputter-based seed metals were uniformly deposited on the sidewall of InP via holes with average θ_2_ values of less than 59 degrees, as shown in Figure 10, and thus the actual R_FOOT_ value is regarded to be a maximum of 3.4.

## 4. Conclusions

Process methodology for sidewall slope control of InP via holes with an etch depth of more than 10 μm for 3D integration was proposed for the first time. The sidewall slope of InP via holes was controlled within the range of 80 to 90 degrees by changing the ICP power in the ICP etcher and utilizing a dry-etched SiO_2_ layer with a sidewall slope of 70 degrees. Furthermore, the sidewall slope of InP via holes was found to be widely adjustable within a range of 36 to 69 degrees, while maintaining high InP etch rate of 1 μm/min, by changing the RF power from 292 W to 306 W and using a wet-etched SiO_2_ layer with a small sidewall slope of 2 degrees; this wide slope control was due to the change of InP-to-SiO_2_ selectivity with RF power. This process methodology for InP via holes is expected to be widely used in implementing InP TSV for 3D integration because of utilization of a common SiO_2_ layer and conventional ICP etcher operated at room temperature and simple gas mixtures of Cl_2_/Ar for InP dry etch.

## Figures and Tables

**Table 1:** Specific process flow and measured results for the SiO_2_ layer with small sidewall slopes.

Process Flow /Measurement	Description	Process Split #1	Process Split #2	Process Split #3	Process Split #4	Process Split #5
Process flow for patterned SiO_2_ layer	SiO_2_ deposition	300 °C in PECVD (UNAXIS SLR-730)				
	Prebake	120 °C, 10 min.				
	HMDS	3000 RPM for 30 s				
	Photoresist (PR)	3000 RPM for 30 s with i-1549 PR				
	Soft bake	90 °C, 90 s				
	Exposure	90 mJ				
	Develop	50 s in MIF 300				
	Hard bake	120 °C, 5 min.	120 °C, 15 min.	120 °C, 15 min.	150 °C, 15 min.	150 °C, 30 min.
	PR Descum	O_2_ of 2500 sccm, pressure of 1 Torr, time of 20 s and room temp. in ICP PR Asher (DAS-2000, PSK, Hwaseong, Korea)				
	Wet-etching	2.5 min. in DHF ^1^	2.5 min. in DHF ^1^	23 min. in BOE ^2^	2.5 min. in DHF ^1^	2.5 min. in DHF ^1^
	PR removal	Acetone—Isopropyl alcohol—DI				
Measurement of patterned SiO_2_ layer	Average thickness (μm)	-	1.02	1.03	1.02	1.02
	Thickness deviation (%)	-	±2.6	±2.7	±2.7	±2.6
	Average sidewall slope (°)	-	2.55	44.25	2	2
	Sidewall slope deviation (%)	-	±5.9	±2.8	±5	±5
	Problem	PR adhesion	-	-	-	-

^1^ DHF: Dilute HF (hydrofluoric acid) solution with a mixture ratio of HF:H_2_O = 1:3; ^2^ BOE: Buffered oxide etch solution with a mixture ratio of NH4F:HF = 38.1 %:2.4 %.

**Table 2:** Footprint comparison of fabricated InP via holes with various sidewall slopes.

Type of Process	InP via Holes with Steep Sidewall Slopes	InP via Holes with Gradual Sidewall Slopes			
Average θ_2_^1^ (°)	80–90	69	59	50	36
W_FOOT_^2^ (μm)	11.5	36	39.2	62.4	101.3
R_FOOT_^3^ (μm/μm)	-	3.1	3.4	5.4	8.8

^1^ θ_2_: The sidewall slope of InP via holes; ^2^ W_FOOT_: The average footprint width of InP via holes; ^3^ R_FOOT_: The W_FOOT_ ratio of InP via holes with gradual sidewall slopes over InP via holes with steep sidewall slopes.

## Data Availability

The data presented in this study are available in article here.
